# Variation in and Hospital Characteristics Associated With the Value of Care for Medicare Beneficiaries With Acute Myocardial Infarction, Heart Failure, and Pneumonia

**DOI:** 10.1001/jamanetworkopen.2018.3519

**Published:** 2018-10-19

**Authors:** Nihar R. Desai, Lesli S. Ott, Elizabeth J. George, Xiao Xu, Nancy Kim, Shengfan Zhou, Angela Hsieh, Sudhakar V. Nuti, Zhenqiu Lin, Susannah M. Bernheim, Harlan M. Krumholz

**Affiliations:** 1 Center for Outcomes Research and Evaluation, Yale–New Haven Hospital, New Haven, Connecticut; 2 Section of Cardiovascular Medicine, Department of Medicine, Yale School of Medicine, New Haven, Connecticut; 3 Remedy Partners, Darien, Connecticut; 4 Department of Medicine, Yale School of Medicine, New Haven, Connecticut; 5 currently with Genentech, South San Francisco, California; 6 currently a student at Yale School of Medicine, New Haven, Connecticut

## Abstract

**Importance:**

Payers and policy makers have advocated for transitioning toward value-based payment models. However, little is known about what is the extent of hospital variation in the value of care and whether there are any hospital characteristics associated with high-value care.

**Objectives:**

To investigate the association between hospital-level 30-day risk-standardized mortality rates (RSMRs) and 30-day risk-standardized payments (RSPs) for acute myocardial infarction (AMI), heart failure (HF), and pneumonia (PNA); to characterize patterns of value in care; and to identify hospital characteristics associated with high-value care (defined by having lower than median RSMRs and RSPs).

**Design, Setting, and Participants:**

This national cross-sectional study applied weighted linear correlation to investigate the association between hospital RSMRs and RSPs for AMI, HF, and PNA between July 1, 2011, and June 30, 2014, among all hospitals; examined correlations in subgroups of hospitals based on key characteristics; and assessed the proportion and characteristics of hospitals delivering high-value care. The data analysis was completed in October 2017. The setting was acute care hospitals. Participants were Medicare fee-for-service beneficiaries discharged with AMI, HF, or PNA.

**Main Outcomes and Measures:**

Hospital-level 30-day RSMRs and RSPs for AMI, HF, and PNA.

**Results:**

The AMI sample consisted of 4339 hospitals with 487 141 hospitalizations for mortality and 462 905 hospitalizations for payment. The HF sample included 4641 hospitals with 960 960 hospitalizations for mortality and 903 721 hospitalizations for payment. The PNA sample contained 4685 hospitals with 952 022 hospitalizations for mortality and 901 764 hospitalizations for payment. The median (interquartile range [IQR]) RSMRs and RSPs, respectively, was 14.3% (IQR, 13.8%-14.8%) and $21 620 (IQR, $20 966-$22 567) for AMI, 11.7% (IQR, 11.0%-12.5%) and $15 139 (IQR, $14 310-$16 118) for HF, and 11.5% (IQR, 10.6%-12.6%) and $14 220 (IQR, $13 342-$15 097) for PNA. There were statistically significant but weak inverse correlations between the RSMRs and RSPs of −0.08 (95% CI, −0.11 to −0.05) for AMI, −0.21 (95% CI, −0.24 to −0.18) for HF, and −0.07 (95% CI, −0.09 to −0.04) for PNA. The largest shared variance between the RSMRs and RSPs was only 4.4% (for HF). The correlations between the RSMRs and RSPs did not differ significantly across teaching status, safety-net status, urban/rural status, or the proportion of patients with low socioeconomic status. Approximately 1 in 4 hospitals (20.9% for AMI, 23.0% for HF, and 23.9% for PNA) had both lower than median RSMRs and RSPs.

**Conclusions and Relevance:**

These findings suggest that there is significant potential for improvement in the value of AMI, HF, and PNA care and also suggest that high-value care for these conditions is attainable across most hospital types.

## Key Points

**Questions:**

What is the variation in the value of care for acute myocardial infarction, heart failure, and pneumonia, and what hospital characteristics are associated with high-value care?

**Findings:**

Among hospitals and patients in this cross-sectional study, there was substantial variation in 30-day risk-standardized mortality and risk-standardized payments, with significant but weak inverse correlations between them. Approximately 1 in 4 hospitals, including facilities across all characteristics, had both lower than median risk-standardized mortality rates and risk-standardized payments for acute myocardial infarction, heart failure, and pneumonia.

**Meaning:**

There appears to be significant opportunity to improve the value of acute myocardial infarction, heart failure, and pneumonia care, and high-value care appears attainable across hospital types.

## Teaser

This cross-sectional study investigates the association between hospital-level 30-day risk-standardized mortality rates and 30-day risk-standardized payments for acute myocardial infarction (MI), heart failure, and pneumonia among Medicare fee-for-service beneficiaries.

## Introduction

Over the past few years, Medicare has dramatically transformed the way it finances health care, aiming to have 90% of fee-for-service payments linked to quality or value from 2008 onward.^1^ More broadly, increasing value in health care has become a top priority for patients, clinicians, payers, and policy makers.^1,2,3,4^ Assessing and ultimately improving value require the measurement of its constituent variables, including health care outcomes achieved and the total cost of resources for a defined and relevant period, often defined as an “episode of care.” However, the association between these 2 domains is poorly understood and limits the ability to meaningfully operationalize the transition from “volume to value.”

A deeper understanding of this association and identification of hospital characteristics associated with higher value and efficiency would have important implications for patients, payers, and policy makers. Whether reductions in resource use can be achieved without compromising the outcomes obtained for patients could illuminate targets for improvements in efficiency and inform alternative payment models. Furthermore, whether the value of care varies as a function of hospital characteristics, geography, and other factors could inform broader discussions about the development and implementation of novel payment programs.

The Centers for Medicare & Medicaid Services (CMS) has publicly reported hospital-level 30-day risk-standardized mortality rates (RSMRs) for acute myocardial infarction (AMI), heart failure (HF), and pneumonia (PNA) for almost 10 years.^5,6,7^ More recently, the CMS began publicly reporting hospital-level 30-day risk-standardized payments (RSPs) for AMI (in 2014) and HF and PNA (in 2015).^8,9,10,11^ These measures, all of which have been endorsed by the National Quality Forum,^12,13,14^ provide a prime opportunity for empirical assessments of the value of care for these clinical conditions.

Accordingly, we examined the association between hospital-level 30-day RSMRs and RSPs for AMI, HF, and PNA to characterize patterns of value in care and to identify hospital characteristics associated with delivery of high-value care. We further assessed the association between these measures for subgroups of hospitals to evaluate if they varied by hospital characteristics.

## Methods

This study was approved by the Human Investigation Committee at the Yale University School of Medicine, New Haven, Connecticut. The use of anonymized administrative data for this study was granted a waiver of informed consent. The Strengthening the Reporting of Observational Studies in Epidemiology (STROBE) reporting guideline for cross-sectional studies was followed.^15^

### Study Cohorts

The study cohorts included hospitalizations of Medicare fee-for-service beneficiaries 65 years or older with a principal discharge diagnosis of AMI, HF, or PNA between July 1, 2011, and June 30, 2014. *International Classification of Diseases*, *Ninth Revision*, *Clinical Modification* diagnosis codes were used to identify the discharges. The data analysis was completed in October 2017.

We defined the study samples consistent with the CMS methods for public reporting, the details of which have been published previously.^5,6,7,8,9,10,11^ The mortality and payment cohorts were restricted to patients enrolled in both Medicare Parts A and B for the 12 months before admission to maximize the ability to risk adjust for patient case mix. Patients who died during the index admission or in the 30-day episode interval were included in both cohorts. However, patients were excluded from the payment cohorts if they were not also enrolled in Medicare Parts A and B during the 30-day episode of care or did not have other sufficient data to capture payments across all acute and postacute care settings and services (eg, lack of a diagnosis related group code for an inpatient hospitalization). For this reason, there are slightly fewer patients in the payment cohorts compared with the mortality cohorts (Table 1). For both cohorts, patients who were discharged against medical advice, had a length of stay of less than 1 day, were transferred to another acute care hospital and discharged with a different principal diagnosis, or were enrolled in hospice at admission or at any time in the previous 12 months were also excluded. For patients with multiple admissions, we included 1 randomly selected admission per patient annually.

**Table 1.** Distribution of RSMRs and RSPs^a^

Variable	No. of Hospitals	No. of Admissions	Mean (SD)	Median (IQR) [Range]
**Acute Myocardial Infarction**				
RSMR, %	4339	487 141	14.3 (1.0)	14.3 (13.8-14.8) [9.9-20.6]
RSP, $	4339	462 905	21 806 (1460)	21 620 (20 966-22 567) [12 862-29 802]
**Heart Failure**				
RSMR, %	4641	960 960	11.7 (1.3)	11.7 (11.0-12.5) [7.2-18.5]
RSP, $	4641	903 721	15 289 (1433)	15 139 (14 310-16 118) [11 086-21 867]
**Pneumonia**				
RSMR, %	4685	952 022	11.6 (1.6)	11.5 (10.6-12.6) [6.9-20.3]
RSP, $	4685	901 764	14 266 (1428)	14 220 (13 342-15 097) [8977-22 999]

Abbreviations: IQR, interquartile range; RSMRs, risk-standardized mortality rates; RSPs, risk-standardized payments.

^a^ Values are inflation adjusted to 2013 US dollars.

### Hospital 30-Day RSMRs and RSPs

We estimated hospital 30-day all-cause RSMRs and 30-day RSPs using methods endorsed by the National Quality Forum and used by the CMS in public reporting.^5,6,7,8,9,10,11^ The analyses of the association between hospital RSMRs and RSPs were limited to hospitals that were present in both the RSMR and RSP measures for each condition. We defined 30-day mortality as death due to any cause within 30 days of the date of admission. Similarly, RSPs capture payments for a 30-day period starting at admission and include payments across all Medicare fee-for-service care settings, services, and supplies except Medicare Part D (this includes inpatient and outpatient care, skilled nursing facilities, home health and hospice care, physician, clinical laboratory, ambulance services, durable medical equipment, prosthetics/orthotics, and other supplies). To isolate payment variation that reflects clinical practice patterns rather than payment policies, payment adjustment factors associated with policy programs (eg, graduate medical education and disproportionate share hospital payment adjustments) were omitted, and adjustment factors associated with zip code were averaged when calculating the individual services that comprise the total payment outcome.^16^ In addition, all payments were inflation adjusted to 2013 US dollars. In calculating RSMR, outcomes for transfer patients were attributed to the first admitting hospital. In calculating RSP, total payment for the 30-day episode of care was attributed to the first admitting hospital.

Hierarchical generalized linear models were constructed to estimate the RSMRs and RSPs for each hospital. The RSMR models were estimated using a binomial distribution and log link, with adjustment for patient age, sex, and 25 clinical covariates for AMI, 22 clinical covariates for HF, and 29 clinical covariates for PNA. The RSP models were estimated using an inverse gaussian distribution and a log link for AMI, a gamma distribution and a log link for HF, and an identity link and a gamma distribution for PNA, as determined by modified Park tests and assessments of model fit. The RSP models were adjusted for patient age and 29 clinical covariates for AMI, 29 clinical covariates for HF, and 47 clinical covariates for PNA. Clinical risk factors were assessed based on diagnosis and procedure codes from claims data during the 12 months before and including the index admission. Codes representing a potential complication of care and found only during the index admission were not adjusted for. Both the mortality and payment models incorporate hospital-level random intercepts to identify hospital-specific random effects and account for the clustering of patients within hospitals. As such, the RSMRs and RSPs reflect the variation in performance across hospitals after accounting for differences in patient clinical characteristics.

### Hospital Characteristics

Our measures of hospital characteristics included teaching status, type of ownership, safety-net status, and urban/rural status based on data from the 2013 American Hospital Association Annual Survey.^17^ We defined safety-net hospitals as either public hospitals or private hospitals with a Medicaid caseload greater than 1 SD above their respective state’s mean private hospital Medicaid caseload, consistent with prior studies.^18,19^ Low socioeconomic status (SES) was defined using the validated Agency for Healthcare Research and Quality (AHRQ) SES index score, a composite of 7 different variables (percentage of people in the labor force who are unemployed, percentage of people living below the poverty level, median household income, median value of owner-occupied dwellings, percentage of people aged ≥25 years with less than a 12th-grade education, percentage of people aged ≥25 years completing ≥4 years of college, and percentage of households that average ≥1 person per room) found in the 2009 to 2013 American Community Survey data.^20^ This score was attributed to each index admission using patients’ 9-digit zip code at the census block group level, and low SES was defined as an AHRQ SES index score less than 42.6755 (the 25th percentile of the AHRQ SES index score). Dual-eligible beneficiaries were defined by having both Medicare and Medicaid coverage during the study interval.

### Statistical Analysis

The baseline characteristics of patients included in both the mortality and payment cohorts were examined overall for AMI, HF, and PNA. Detailed payments across care settings and services were examined overall for AMI, HF, and PNA after excluding patients with outlier payments (winsorization). We then assessed the distributions of the RSMRs and RSPs across all eligible hospitals in our sample and examined linear and nonlinear associations between the RSMRs and RSPs for each condition. Specifically, we calculated the Pearson product moment correlation coefficient between the estimated RSMRs and RSPs weighted by the hospital mean of RSMR and RSP volumes, given that each measure has uncertainty based on the observed number of cases, as well as the degree of within-hospital clustering. The percentage of variance in RSP accounted for by RSMR (ie, the shared variance) was calculated for AMI, HF, and PNA using both the square of the correlation coefficient (*r^2^*) and the deviance explained for generalized additive models. To identify potential nonlinear associations between RSMRs and RSPs, we fit generalized additive models for each condition using RSMR as the dependent variable and a cubic spline smoother of RSP as the independent variable. The correlation was also assessed by applying Mantel-Haenszel χ^2^ testing within different hospital groups stratified by key characteristics (eg, teaching vs nonteaching).

For each condition, we classified hospitals as having a high vs low RSMR and RSP based on whether their corresponding values were above or equal to vs below the respective sample median. We considered hospitals to be providing high-value care if both their RSMRs and RSPs were lower than the sample median values. We then examined hospital and patient characteristics across the different combinations of RSMR and RSP categories (ie, low RSMR: low RSP, low RSMR: high RSP, high RSMR: low RSP, and high RSMR: high RSP) to investigate whether there was a particular set of characteristics associated with delivery of high-value care. As a sensitivity analysis, hospitals were further stratified into high-value vs low-value groups based on the highest and lowest quartiles (instead of medians) of both the RSMR and RSP for each condition to create a more restrictive definition of value, and comparison of hospital characteristics across these groups was repeated using Mantel-Haenszel χ^2^ testing.

We used the mgcv package in R (The R Project for Statistical Computing) to fit generalized additive models. All other analyses were conducted using SAS version 9.3 (SAS Institute Inc). All tests of statistical significance were 2-tailed and evaluated at a significance level of .05, corrected for multiple comparisons using the correction by Šidák.^21^

## Results

### Study Sample

The AMI sample consisted of 4339 hospitals with 487 141 hospitalizations for mortality and 462 905 hospitalizations for payment (Table 1). The HF sample included 4641 hospitals with 960 960 hospitalizations for mortality and 903 721 hospitalizations for payment. The PNA sample contained 4685 hospitals with 952 022 hospitalizations for mortality and 901 764 hospitalizations for payment. The RSMRs and RSPs for all 3 conditions varied widely. For AMI, RSMRs ranged from 9.9% to 20.6% (median, 14.3%; interquartile range [IQR], 13.8%-14.8%); RSPs ranged from $12 862 to $29 802 (median, $21 620; IQR, $20 966-$22 567). For HF, RSMRs ranged from 7.2% to 18.5% (median, 11.7%; IQR, 11.0%-12.5%); RSPs ranged from $11 086 to $21 867 (median, $15 139; IQR, $14 310-$16 118). For PNA, RSMRs ranged from 6.9% to 20.3% (median, 11.5%; IQR, 10.6%-12.6%); RSPs ranged from $8977 to $22 999 (median, $14 220; IQR, $13 342-$15 097). The baseline patient characteristics for the AMI, HF, and PNA cohorts are listed in eTables 1, 2, and 3 in the Supplement, respectively. Detailed payment data by site of care and service for each clinical cohort are listed in eTables 4, 5, and 6 in the Supplement. While the index hospitalization accounted for most of the 30-day RSPs, there was substantially more variation in postacute care spending for all 3 conditions. Specifically, there was almost 10-fold variation in the median (IQR) spending on readmissions and skilled nursing facility use for AMI, HF, and PNA.

### Association Between RSMRs and RSPs

For the overall sample, there were statistically significant, inverse associations between RSMRs and RSPs for AMI, HF, and PNA (Figure and Table 2). The strength of the association was modest, with a Pearson product moment correlation coefficient (*r*) of −0.08 (95% CI, −0.11 to −0.05) for AMI, −0.21 (95% CI, −0.24 to −0.18) for HF, and −0.07 (95% CI, −0.09 to −0.04) for PNA. The largest shared variance (*r^2^*) between RSMRs and RSPs was only 4.4% (for HF). The results from generalized additive models were consistent with the findings of a weak association between RSMRs and RSPs (the deviance explained was 2.7%, 4.1%, and 0.6% for AMI, HF, and PNA, respectively). In subgroup analyses, the correlations between RSMRs and RSPs did not differ substantially across subgroups of hospital types, including teaching status, type of ownership, safety-net status, urban/rural status, number of beds, and the proportions of patients who are Medicaid beneficiaries, black, low SES, or dually eligible for Medicare and Medicaid (Table 2).

**Figure.**
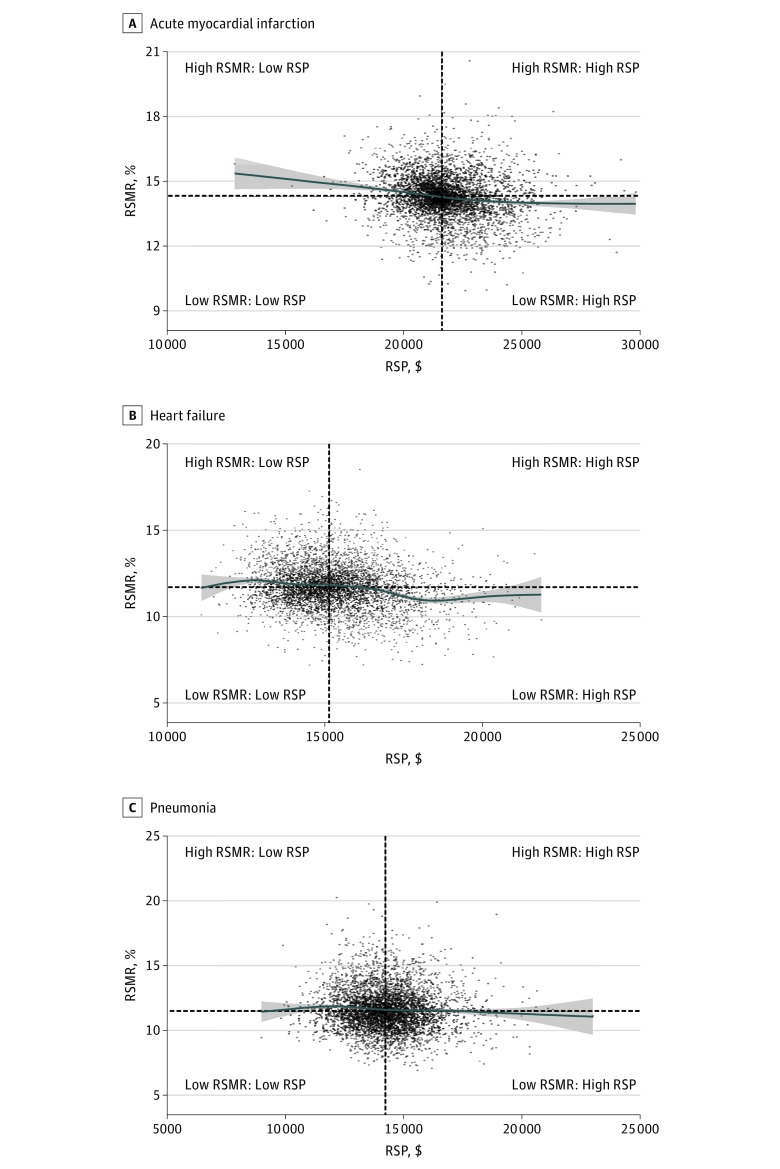
Scatterplot of Hospital-Level RSMRs and RSPs Values are inflation adjusted to 2013 US dollars. Blue lines show the cubic spline smooth regression lines, with risk-standardized mortality rates (RSMRs) as the dependent variable and risk-standardized payments (RSPs) as the independent variable. Tinted areas around the cubic spline regression lines show the 95% CIs. The Pearson product moment correlation coefficients are −0.08 (95% CI, −0.11 to −0.05) for acute myocardial infarction (n = 4339) (A), −0.21 (95% CI, −0.24 to −0.18) for heart failure (n = 4641) (B), and −0.07 (95% CI, −0.09 to −0.04) for pneumonia (n = 4685) (C). The horizontal and vertical dotted lines indicate the median RSMR and RSP, respectively.

**Table 2.** Pearson Product Moment Correlation of Risk-Standardized Mortality Rates and Risk-Standardized Payments for Acute Myocardial Infarction, Heart Failure, and Pneumonia Overall and Across Hospital Characteristics^a^

Variable	Acute Myocardial Infarction		Heart Failure		Pneumonia	
	No. of Hospitals	Correlation (95% CI)	No. of Hospitals	Correlation (95% CI)	No. of Hospitals	Correlation (95% CI)
All hospitals	4339	−0.08 (−0.11 to −0.05)	4641	−0.21 (−0.24 to −0.18)	4685	−0.07 (−0.09 to −0.04)
Teaching status						
Teaching	846	−0.09 (−0.15 to −0.02)	861	−0.16 (−0.23 to −0.10)	862	−0.01 (−0.08 to 0.05)
Nonteaching	3383	−0.07 (−0.10 to −0.03)	3644	−0.20 (−0.23 to −0.17)	3681	−0.05 (−0.08 to −0.01)
Type of ownership						
Private for-profit	696	−0.06 (−0.14 to 0.01)	743	−0.16 (−0.23 to −0.09)	753	−0.05 (−0.12 to 0.02)
Private not-for-profit	2626	−0.08 (−0.12 to −0.04)	2733	−0.22 (−0.25 to −0.18)	2739	−0.07 (−0.11 to −0.03)
Public	907	−0.10 (−0.16 to −0.03)	1029	−0.14 (−0.20 to −0.08)	1051	−0.05 (−0.11 to 0.01)
Safety-net status						
Safety-net hospital	1216	−0.07 (−0.13 to −0.02)	1361	−0.20 (−0.25 to −0.15)	1385	−0.10 (−0.15 to −0.04)
Non–safety-net hospital	3013	−0.08 (−0.11 to −0.04)	3144	−0.21 (−0.24 to −0.17)	3158	−0.04 (−0.08 to −007)
Urban/rural status						
Urban	3222	−0.08 (−0.11 to −0.04)	3340	−0.20 (−0.24 to −0.17)	3363	−0.05 (−0.09 to −0.02)
Rural	1007	−0.06 (−0.00 to −0.12)	1165	−0.05 (−0.11 to −0.01)	1180	0.01 (−0.05 to 0.07)
No. of beds						
1-199	2900	−0.13 (−0.16 to −0.09)	3170	−0.17 (−0.20 to −0.13)	3204	−0.06 (−0.10 to −0.03)
200-299	539	−0.04 (−0.13 to 0.04)	542	−0.17 (−0.25 to −0.08)	543	0.01 (−0.08 to 0.09)
300-399	311	0.00 (−0.11 to 0.11)	312	−0.13 (−0.23 to −0.01)	313	0.21 (0.10 to 0.31)
400-499	175	−0.15 (−0.29 to 0.00)	176	−0.24 (−0.37 to −0.09)	177	−0.02 (−0.16 to 0.13)
≥500	270	−0.04 (−0.16 to 0.08)	271	−0.08 (−0.2 to 0.04)	272	0.02 (−0.10 to 0.14)
Proportion of patients who are Medicaid beneficiaries, %						
0-10	1006	−0.13 (−0.19 to −0.07)	1160	−0.20 (−0.25 to −0.14)	1188	−0.03 (−0.08 to 0.03)
11-20	1931	−0.07 (−0.12 to −0.03)	2025	−0.20 (−0.24 to −0.16)	2028	−0.05 (−0.10 to −0.01)
21-30	891	−0.02 (−0.09 to 0.04)	911	−0.21 (−0.27 to −0.15)	913	−0.07 (−0.13 to 0.00)
>30	367	−0.13 (−0.23 to −0.03)	375	−0.24 (−0.33 to −0.14)	380	−0.14 (−0.24 to −0.04)
Proportion of patients who are black						
Quartile 1, lowest proportion	574	−0.06 (−0.14 to 0.02)	721	−0.17 (−0.24 to −0.10)	725	−0.08 (−0.15 to −0.01)
Quartile 2	576	−0.11 (−0.19 to −0.02)	717	−0.24 (−0.31 to −0.17)	723	−0.10 (−0.17 to −0.02)
Quartile 3	566	−0.06 (−0.14 to 0.02)	720	−0.22 (−0.29 to −0.15)	724	0.02 (−0.05 to 0.09)
Quartile 4, highest proportion	572	−0.05 (−0.13 to 0.03)	717	−0.16 (−0.23 to −0.09)	724	−0.04 (−0.11 to 0.04)
Hospitals with 0 black patients	1941	−0.12 (−0.17 to −0.08)	1630	−0.24 (−0.28 to −0.19)	1647	−0.08 (−0.13 to −0.03)
Proportion of patients with low SES						
Quartile 1, lowest proportion	738	−0.15 (−0.22 to −0.07)	892	−0.22 (−0.28 to −0.15)	952	−0.05 (−0.12 to 0.01)
Quartile 2	735	−0.09 (−0.16 to −0.01)	891	−0.19 (−0.26 to −0.13)	951	0.00 (−0.07 to 0.06)
Quartile 3	735	0.04 (−0.04 to 0.11)	892	−0.16 (−0.22 to −0.09)	952	−0.04 (−0.10 to 0.02)
Quartile 4, highest proportion	735	−0.09 (−0.16 to −0.02)	891	−0.30 (−0.36 to −0.24)	951	−0.12 (−0.18 to −0.06)
Hospitals with 0 patients with low SES	1286	−0.28 (−0.33 to −0.23)	939	−0.31 (−0.37 to −0.26)	737	−0.23 (−0.29 to −0.16)
Proportion of patients who are dually eligible for Medicare and Medicaid						
Quartile 1, lowest proportion	916	−0.09 (−0.15 to −0.03)	1073	−0.21 (−0.26 to −0.15)	1105	−0.01 (−0.07 to 0.05)
Quartile 2	916	−0.08 (−0.14 to −0.02)	1079	−0.17 (−0.22 to −0.11)	1098	−0.02 (−0.08 to 0.04)
Quartile 3	997	0.02 (−0.04 to 0.08)	1066	−0.16 (−0.22 to −0.10)	1123	−0.02 (−0.08 to 0.04)
Quartile 4, highest proportion	832	−0.2 (−0.26 to −0.13)	1072	−0.37 (−0.42 to −0.31)	1077	−0.18 (−0.23 to −0.12)
Hospitals with 0 dually eligible patients	568	−0.37 (−0.44 to −0.3)	215	−0.64 (−0.71 to −0.55)	140	−0.53 (−0.64 to −0.40)

Abbreviation: SES, socioeconomic status.

^a^ For acute myocardial infarction, 4229 of 4339 hospitals (97.5%) were matched to American Hospital Association data. For heart failure, 4505 of 4641 hospitals (97.1%) were matched to American Hospital Association data. For pneumonia, 4543 of 4685 hospitals (97.0%) were matched to American Hospital Association data.

### Value of Care and Hospital Characteristics

Overall, approximately 1 in 4 hospitals (20.9% for AMI, 23.0% for HF, and 23.9% for PNA) had both lower than median RSMRs and RSPs. Patient characteristics for each clinical condition across the 4 value groups based on the median RSMR and RSP (ie, low RSMR: low RSP, low RSMR: high RSP, high RSMR: low RSP, and high RSMR: high RSP) are listed in eTables 1, 2, and 3 in the Supplement. There were no clinically relevant differences in the baseline characteristics across hospital groups. Detailed payment data by site of care and service for each clinical cohort across groups stratified by the median RSMR and RSP are listed in eTables 4, 5, and 6 in the Supplement. Hospitals with higher than median RSP for AMI, HF, and PNA had greater spending for the index hospitalization, postacute care, and all other services, regardless of performance on RSMR (low or high). Hospitals with low RSMR and low RSP for AMI, HF, and PNA had significantly lower readmissions and markedly lower use of nonacute inpatient care across conditions.

Hospital characteristics for each clinical condition across the 4 value groups based on the median RSMR and RSP are listed in Table 3. There were significant differences in hospital profiles across groups for all clinical conditions. Hospitals in the low RSMR: high RSP group were more likely to be larger, teaching hospitals, private ownership, non–safety-net hospitals, and urban hospitals, while hospitals in the high RSMR: low RSP group were more likely to be smaller, nonteaching hospitals, public ownership, safety-net hospitals, and rural hospitals. There was no consistent trend in grouping of RSMR and RSP based on the proportions of patients who are Medicaid beneficiaries, black, low SES, or dually eligible for Medicare and Medicaid.

**Table 3.** Number of Hospitals in Categories of RSMRs and RSPs by Hospital Characteristics (Low or High Designation Based on the Median)^a^

Variable	No. (%)					*P* Value
	Low RSMR		High RSMR		Total	
	Low RSP	High RSP	Low RSP	High RSP		
**Acute Myocardial Infarction**						
No. of hospitals	886	1222	1229	892	4229	NA
RSMR, median (IQR), %	13.9 (13.4-14.1)	13.7 (13.1-14.0)	14.8 (14.5-15.2)	14.8 (14.5-15.4)	NA	NA
RSP, median (IQR), $	21 009 (20 393-21 379)	22 621 (22 005-23 483)	20 925 (20 320-21 319)	22 540 (21 992-23 408)	NA	NA
Teaching status						
Teaching	163 (19.3)	329 (38.9)	130 (15.4)	224 (26.5)	846 (100)	<.001
Nonteaching	723 (21.4)	893 (26.4)	1099 (32.4)	668 (19.7)	3383 (100)	
Type of ownership						
Private for-profit	129 (18.5)	219 (31.5)	167 (24.0)	181 (26.0)	696 (100)	<.001
Private not-for-profit	554 (21.0)	809 (30.8)	708 (27.0)	555 (21.1)	2626 (100)	
Public	203 (22.4)	194 (21.4)	354 (39.0)	156 (17.2)	907 (100)	
Safety-net status						
Safety-net hospital	270 (22.2)	284 (23.4)	446 (36.7)	216 (17.8)	1216 (100)	<.001
Non–safety-net hospital	616 (20.4)	938 (31.1)	783 (26.0)	676 (22.4)	3013 (100)	
Urban/rural status						
Urban	654 (20.3)	1026 (31.8)	809 (25.1)	733 (22.7)	3222 (100)	<.001
Rural	232 (23.0)	196 (19.5)	420 (41.7)	159 (15.8)	1007 (100)	
No. of beds						
1-199	641 (22.0)	713 (24.4)	1027 (35.2)	538 (18.4)	2919 (100)	<.001
200-299	106 (19.6)	200 (37.0)	91 (16.8)	144 (26.6)	541 (100)	
300-399	58 (18.4)	117 (37.0)	55 (17.4)	86 (27.2)	316 (100)	
400-499	28 (15.9)	70 (39.8)	26 (14.8)	52 (29.5)	176 (100)	
≥500	51 (18.7)	122 (44.7)	30 (11.0)	70 (25.6)	273 (100)	
Proportion of patients who are Medicaid beneficiaries, %						
0-10	201 (19.9)	316 (31.3)	316 (31.3)	175 (17.4)	1008 (100)	.002
11-20	432 (22.2)	552 (28.4)	555 (28.5)	405 (20.8)	1944 (100)	
21-30	169 (18.7)	251 (27.8)	250 (27.7)	233 (25.8)	903 (100)	
>30	82 (22.2)	103 (27.8)	108 (29.2)	77 (20.8)	370 (100)	
Proportion of patients who are black						
Quartile 1, lowest proportion	111 (19.2)	229 (39.6)	99 (17.1)	139 (24.0)	578 (100)	<.001
Quartile 2	94 (16.3)	237 (41.0)	110 (19.0)	137 (23.7)	578 (100)	
Quartile 3	110 (19.0)	181 (31.3)	116 (20.1)	171 (29.6)	578 (100)	
Quartile 4, highest proportion	138 (23.9)	153 (26.5)	165 (28.5)	122 (21.1)	578 (100)	
Hospitals with 0 black patients	433 (22.6)	422 (22.0)	739 (38.5)	323 (16.8)	1917 (100)	
Proportion of patients with low SES						
Quartile 1, lowest proportion	144 (19.4)	279 (37.6)	161 (21.7)	159 (21.4)	743 (100)	
Quartile 2	140 (18.9)	271 (36.7)	165 (22.3)	163 (22.1)	739 (100)	
Quartile 3	147 (19.8)	226 (30.5)	166 (22.4)	202 (27.3)	741 (100)	
Quartile 4, highest proportion	183 (24.7)	151 (20.4)	262 (35.4)	145 (19.6)	741 (100)	
Hospitals with 0 patients with low SES	272 (21.5)	295 (23.3)	475 (37.5)	223 (17.6)	1265 (100)	
Proportion of patients who are dually eligible for Medicare and Medicaid						
Quartile 1, lowest proportion	178 (19.2)	353 (38.1)	186 (20.1)	210 (22.7)	927 (100)	<.001
Quartile 2	180 (19.6)	277 (30.1)	236 (25.7)	227 (24.7)	920 (100)	
Quartile 3	220 (21.9)	243 (24.2)	319 (31.7)	224 (22.3)	1006 (100)	
Quartile 4, highest proportion	193 (23.1)	212 (25.3)	281 (33.6)	151 (18.0)	837 (100)	
Hospitals with 0 dually eligible patients	115 (21.3)	137 (25.4)	207 (38.4)	80 (14.8)	539 (100)	
**Heart Failure**						
No. of hospitals	1037	1207	1212	1049	4505	NA
RSMR, median (IQR), %	11.1 (10.6-11.4)	10.9 (10.1-11.3)	12.5 (12.1-13.2)	12.4 (12.0-13.1)	NA	NA
RSP, median (IQR), $	14 320 (13 804-14 762)	16 256 (15 699-17 226)	14 274 (13 670-14 720)	15 984 (15 522-16 610)	NA	NA
Teaching status						
Teaching	151 (17.5)	374 (43.4)	111 (12.9)	225 (26.1)	861 (100)	<.001
Nonteaching	886 (24.3)	833 (22.9)	1101 (30.2)	824 (22.6)	3644 (100)	
Type of ownership						
Private for-profit	159 (21.4)	249 (33.5)	165 (22.2)	170 (22.9)	743 (100)	<.001
Private not-for-profit	591 (21.6)	757 (27.7)	728 (26.6)	657 (24.0)	2733 (100)	
Public	287 (27.9)	201 (19.5)	319 (31.0)	222 (21.6)	1029 (100)	
Safety-net status						
Safety-net hospital	367 (27.0)	303 (22.2)	408 (30.0)	283 (20.8)	1361 (100)	<.001
Non–safety-net hospital	670 (21.3)	904 (28.8)	804 (25.6)	766 (24.4)	3144 (100)	
Urban/rural status						
Urban	727 (21.8)	1019 (30.5)	796 (23.8)	798 (23.9)	3340 (100)	<.001
Rural	310 (26.6)	188 (16.1)	416 (35.7)	251 (21.5)	1165 (100)	
No. of beds						
1-199	861 (27.0)	634 (19.9)	1029 (32.3)	665 (20.9)	3189 (100)	<.001
200-299	84 (15.4)	196 (36.0)	93 (17.1)	171 (31.4)	544 (100)	
300-399	36 (11.4)	146 (46.1)	43 (13.6)	92 (29.0)	317 (100)	
400-499	19 (10.7)	81 (45.8)	29 (16.4)	48 (27.1)	177 (100)	
≥500	37 (13.5)	148 (54.0)	16 (5.8)	73 (26.6)	274 (100)	
Proportion of patients who are Medicaid beneficiaries, %						
0-10	277 (23.8)	307 (26.4)	306 (26.3)	272 (23.4)	1162 (100)	.01
11-20	458 (22.5)	512 (25.1)	560 (27.5)	508 (24.9)	2038 (100)	
21-30	208 (22.5)	262 (28.4)	244 (26.4)	209 (22.6)	923 (100)	
>30	94 (24.9)	124 (32.8)	100 (26.5)	60 (15.9)	378 (100)	
Proportion of patients who are black						
Quartile 1, lowest proportion	132 (18.2)	187 (25.8)	202 (27.8)	205 (28.2)	726 (100)	<.001
Quartile 2	128 (17.6)	233 (32.1)	156 (21.5)	209 (28.8)	726 (100)	
Quartile 3	130 (17.9)	281 (38.7)	138 (19.0)	177 (24.4)	726 (100)	
Quartile 4, highest proportion	212 (29.2)	248 (34.2)	145 (20.0)	120 (16.6)	725 (100)	
Hospitals with 0 black patients	435 (27.2)	258 (16.1)	571 (35.6)	338 (21.1)	1602 (100)	
Proportion of patients with low SES						
Quartile 1, lowest proportion	169 (18.8)	248 (27.6)	260 (28.9)	222 (24.7)	899 (100)	<.001
Quartile 2	186 (20.7)	237 (26.4)	236 (26.3)	239 (26.6)	898 (100)	
Quartile 3	183 (20.4)	311 (34.6)	168 (18.7)	236 (26.3)	898 (100)	
Quartile 4, highest proportion	280 (31.2)	233 (25.9)	247 (27.5)	138 (15.4)	898 (100)	
Hospitals with 0 patients with low SES	219 (24.0)	178 (19.5)	301 (33.0)	214 (23.5)	912 (100)	
Proportion of patients who are dually eligible for Medicare and Medicaid	203 (18.8)	311 (28.8)	282 (26.1)	284 (26.3)	1080 (100)	
Quartile 1, lowest proportion	209 (19.4)	278 (25.7)	308 (28.5)	285 (26.4)	1080 (100)	<.001
Quartile 2	233 (21.6)	278 (25.7)	294 (27.2)	275 (25.5)	1080 (100)	
Quartile 3	341 (31.6)	289 (26.8)	271 (25.1)	179 (16.6)	1080 (100)	
Quartile 4, highest proportion	51 (27.6)	51 (27.6)	57 (30.8)	26 (14.1)	185 (100)	
Hospitals with 0 dually eligible patients						
**Pneumonia**						
No. of hospitals	1084	1181	1170	1108	4543	NA
RSMR, median (IQR), %	10.6 (10.0-11.1)	10.5 (9.8-11.0)	12.6 (12.0-13.6)	12.5 (11.9-13.4)	NA	NA
RSP, median (IQR), $	13 370 (12 757-13 815)	15 087 (14 631-15 821)	13 317 (12 649-13 797)	15 118 (14 649-15 774)	NA	NA
Teaching status						
Teaching	190 (22.0)	344 (39.9)	118 (13.7)	210 (24.4)	862 (100)	<.001
Nonteaching	894 (24.3)	837 (22.7)	1052 (28.6)	898 (24.4)	3681 (100)	
Type of ownership						
Private for-profit	161 (21.4)	226 (30.0)	165 (21.9)	201 (26.7)	753 (100)	<.001
Private not-for-profit	672 (24.5)	764 (27.9)	669 (24.4)	634 (23.1)	2739 (100)	
Public	251 (23.9)	191 (18.2)	336 (32.0)	273 (26.0)	1051 (100)	
Safety-net status						
Safety-net hospital	322 (23.2)	272 (19.6)	452 (32.6)	339 (24.5)	1385 (100)	<.001
Non–safety-net hospital	762 (24.1)	909 (28.8)	718 (22.7)	769 (24.4)	3158 (100)	
Urban/rural status						
Urban	778 (23.1)	996 (29.6)	767 (22.8)	822 (24.4)	3363 (100)	<.001
Rural	306 (25.9)	185 (15.7)	403 (34.2)	286 (24.2)	1180 (100)	
No. of beds						
1-199	834 (25.9)	667 (20.7)	1001 (31.1)	721 (22.4)	3223 (100)	<.001
200-299	108 (19.8)	181 (33.2)	93 (17.1)	163 (29.9)	545 (100)	
300-399	50 (15.7)	124 (39.0)	40 (12.6)	104 (32.7)	318 (100)	
400-499	38 (21.3)	73 (41.0)	20 (11.2)	47 (26.4)	178 (100)	
≥500	54 (19.6)	134 (48.7)	16 (5.8)	71 (25.8)	275 (100)	
Proportion of patients who are Medicaid beneficiaries, %						
0-10	310 (26.1)	303 (25.5)	282 (23.7)	295 (24.8)	1190 (100)	<.001
11-20	489 (24.0)	524 (25.7)	521 (25.5)	507 (24.8)	2041 (100)	
21-30	196 (21.2)	247 (26.7)	267 (28.9)	215 (23.2)	925 (100)	
>30	89 (23.2)	105 (27.4)	100 (26.1)	89 (23.2)	383 (100)	
Proportion of patients who are black						
Quartile 1, lowest proportion	169 (23.1)	193 (26.4)	206 (28.2)	163 (22.3)	731 (100)	.19
Quartile 2	147 (20.1)	237 (32.4)	161 (22.0)	186 (25.4)	731 (100)	
Quartile 3	141 (19.3)	270 (36.9)	117 (16.0)	203 (27.8)	731 (100)	
Quartile 4, highest proportion	166 (22.7)	207 (28.3)	156 (21.3)	202 (27.6)	731 (100)	
Hospitals with 0 black patients	461 (28.5)	274 (16.9)	530 (32.7)	354 (21.9)	1619 (100)	
Proportion of patients with low SES						
Quartile 1, lowest proportion	237 (24.7)	267 (27.8)	240 (25.0)	215 (22.4)	959 (100)	<.001
Quartile 2	217 (22.6)	272 (28.3)	238 (24.8)	233 (24.3)	960 (100)	
Quartile 3	204 (21.3)	297 (31.0)	199 (20.8)	258 (26.9)	958 (100)	
Quartile 4, highest proportion	238 (24.8)	203 (21.2)	292 (30.5)	225 (23.5)	958 (100)	
Hospitals with 0 patients with low SES	188 (26.6)	142 (20.1)	201 (28.4)	177 (25.0)	708 (100)	
Proportion of patients who are dually eligible for Medicare and Medicaid						
Quartile 1, lowest proportion	292 (26.3)	341 (30.7)	226 (20.4)	250 (22.5)	1109 (100)	<.001
Quartile 2	249 (22.3)	282 (25.3)	301 (27.0)	283 (25.4)	1115 (100)	
Quartile 3	245 (21.8)	264 (23.5)	306 (27.2)	309 (27.5)	1124 (100)	
Quartile 4, highest proportion	261 (24.1)	271 (25.0)	304 (28.0)	249 (22.9)	1085 (100)	
Hospitals with 0 dually eligible patients	37 (33.6)	23 (20.9)	33 (30.0)	17 (15.5)	110 (100)	

Abbreviations: IQR, interquartile range; NA, not applicable; RSMRs, risk-standardized mortality rates; RSPs, risk-standardized payments; SES, socioeconomic status.

^a^ Values are inflation adjusted to 2013 US dollars. For acute myocardial infarction, 4229 of 4339 hospitals (97.5%) were matched to American Heart Association data. For heart failure, 4505 of 4641 hospitals (97.1%) were matched to American Heart Association data. For pneumonia, 4543 of 4685 hospitals (97.0%) were matched to American Heart Association data. Percentages may not sum to 100 because of rounding. Data are number (percentage) unless otherwise indicated.

Regardless of size, setting, teaching status, safety-net status, and the proportions of patients from vulnerable populations, hospitals were able to achieve low RSMRs and RSPs for AMI, HF, and PNA. For example, across all 3 conditions, approximately 20% of both teaching and nonteaching hospitals were classified as delivering high-value care. Similarly, just over 20% of both safety-net and non–safety-net hospitals achieved high-value care. Approximately 25% of hospitals with the highest proportions of patients who are Medicaid beneficiaries, black, low SES, or dually eligible for Medicare and Medicaid achieved low RSMR and low RSR for AMI, HF, and PNA.

A sensitivity analysis for hospital characteristics across the more restrictive value groups based on quartiles of both the RSMR and RSP produced similar patterns observed for the analysis based on the median values of RSMR and RSP. These results are shown in Table 4.

**Table 4.** Number of Hospitals in Categories of RSMRs and RSPs by Hospital Characteristics (Low or High Designation Based on Quartiles)^a^

Variable	No. (%)					*P* Value
	Low RSMR		High RSMR		Total	
	Low RSP	High RSP	Low RSP	High RSP		
**Acute Myocardial Infarction**						
No. of hospitals	219	443	396	261	1319	NA
RSMR, median (IQR), %	13.4 (12.9-13.6)	13.2 (12.6-13.5)	15.3 (15.0-15.8)	15.4 (15.1-16.0)	NA	NA
RSP, median (IQR), $	20 305 (19 736-20 666)	23 472 (22 992-24 303)	20 255 (19 667-20 608)	23 448 (22 953-24 286)	NA	NA
Teaching status						
Teaching	53 (14.5)	167 (45.8)	58 (15.9)	87 (23.8)	365 (100)	<.001
Nonteaching	166 (17.4)	276 (28.9)	338 (35.4)	174 (18.2)	954 (100)	
Type of ownership						
Private for-profit	32 (13.7)	96 (41.2)	55 (23.6)	50 (21.5)	233 (100)	<.001
Private not-for-profit	155 (17.3)	321 (35.7)	240 (26.7)	182 (20.3)	898 (100)	
Public	32 (17.0)	26 (13.8)	101 (53.7)	29 (15.4)	188 (100)	
Safety-net status						
Safety-net hospital	53 (18.2)	56 (19.2)	136 (46.6)	47 (16.1)	292 (100)	<.001
Non–safety-net hospital	166 (16.2)	387 (37.7)	260 (25.3)	214 (20.8)	1027 (100)	
Urban/rural status						
Urban	195 (16.9)	435 (37.7)	282 (24.4)	243 (21.0)	1155 (100)	<.001
Rural	24 (14.6)	8 (4.9)	114 (69.5)	18 (11.0)	164 (100)	
No. of beds						
1-199	130 (18.2)	169 (23.6)	307 (42.9)	109 (15.2)	715 (100)	<.001
200-299	43 (17.4)	113 (45.7)	32 (13.0)	59 (23.9)	247 (100)	
300-399	17 (12.1)	58 (41.1)	28 (19.9)	38 (27.0)	141 (100)	
400-499	10 (13.0)	31 (40.3)	12 (15.6)	24 (31.2)	77 (100)	
≥500	19 (13.7)	72 (51.8)	17 (12.2)	31 (22.3)	139 (100)	
Proportion of patients who are Medicaid beneficiaries, %						
0-10	31 (13.8)	97 (43.1)	60 (26.7)	37 (16.4)	225 (100)	.05
11-20	124 (18.9)	203 (30.9)	205 (31.2)	125 (19.0)	657 (100)	
21-30	42 (13.8)	102 (33.6)	92 (30.3)	68 (22.4)	304 (100)	
>30	22 (16.5)	41 (30.8)	39 (29.3)	31 (23.3)	133 (100)	
Proportion of patients who are black						
Quartile 1, lowest proportion	43 (17.1)	117 (46.4)	45 (17.9)	47 (18.7)	252 (100)	<.001
Quartile 2	24 (9.3)	115 (44.7)	53 (20.6)	65 (25.3)	257 (100)	
Quartile 3	37 (16.4)	85 (37.6)	39 (17.3)	65 (28.8)	226 (100)	
Quartile 4, highest proportion	35 (17.8)	60 (30.5)	59 (29.9)	43 (21.8)	197 (100)	
Hospitals with 0 black patients	80 (20.7)	66 (17.1)	200 (51.7)	41 (10.6)	387 (100)	
Proportion of patients with low SES						
Quartile 1, lowest proportion	49 (15.3)	137 (42.7)	76 (23.7)	59 (18.4)	321 (100)	<.001
Quartile 2	46 (15.4)	118 (39.5)	67 (22.4)	68 (22.7)	299 (100)	
Quartile 3	48 (17.4)	97 (35.1)	64 (23.2)	67 (24.3)	276 (100)	
Quartile 4, highest proportion	35 (17.2)	48 (23.5)	81 (39.7)	40 (19.6)	204 (100)	
Hospitals with 0 patients with low SES	41 (18.7)	43 (19.6)	108 (49.3)	27 (12.3)	219 (100)	
Proportion of patients who are dually eligible for Medicare and Medicaid						
Quartile 1, lowest proportion	64 (16.2)	170 (43.0)	80 (20.3)	81 (20.5)	395 (100)	<.001
Quartile 2	53 (15.6)	117 (34.4)	102 (30.0)	68 (20.0)	340 (100)	
Quartile 3	58 (17.2)	91 (26.9)	110 (32.5)	79 (23.4)	338 (100)	
Quartile 4, highest proportion	36 (17.6)	61 (29.9)	75 (36.8)	32 (15.7)	204 (100)	
Hospitals with 0 dually eligible patients	8 (19.0)	4 (9.5)	29 (69.0)	1 (2.4)	42 (100)	
**Heart Failure**						
No. of hospitals	231	420	338	223	1212	NA
RSMR, median (IQR), %	10.6 (10.1-10.8)	10.1 (9.6-10.7)	13.2 (12.8-13.9)	13.1 (12.7-13.7)	NA	NA
RSP, median (IQR), $	13 783 (13 350-14 050)	17 211 (16 617-17 916)	13 624 (13 157-13 977)	16 713 (16 342-17 287)	NA	NA
Teaching status						
Teaching	33 (12.3)	161 (60.1)	19 (7.1)	55 (20.5)	268 (100)	<.001
Nonteaching	198 (21.0)	259 (27.4)	319 (33.8)	168 (17.8)	944 (100)	
Type of ownership						
Private for-profit	37 (15.2)	106 (43.6)	45 (18.5)	55 (22.6)	243 (100)	<.001
Private not-for-profit	136 (18.8)	269 (37.1)	199 (27.4)	121 (16.7)	725 (100)	
Public	58 (23.8)	45 (18.4)	94 (38.5)	47 (19.3)	244 (100)	
Safety-net status						
Safety-net hospital	83 (24.8)	77 (23.0)	117 (34.9)	58 (17.3)	335 (100)	<.001
Non–safety-net hospital	148 (16.9)	343 (39.1)	221 (25.2)	165 (18.8)	877 (100)	
Urban/rural status						
Urban	172 (17.8)	398 (41.1)	215 (22.2)	183 (18.9)	968 (100)	<.001
Rural	59 (24.2)	22 (9.0)	123 (50.4)	40 (16.4)	244 (100)	
No. of beds						
1-199	181 (23.7)	161 (21.0)	307 (40.1)	116 (15.2)	765 (100)	<.001
200-299	24 (13.9)	84 (48.6)	20 (11.6)	45 (26.0)	173 (100)	
300-399	9 (8.5)	65 (61.3)	5 (4.7)	27 (25.5)	106 (100)	
400-499	6 (10.9)	35 (63.6)	4 (7.3)	10 (18.2)	55 (100)	
≥500	11 (9.7)	75 (66.4)	2 (1.8)	25 (22.1)	113 (100)	
Proportion of patients who are Medicaid beneficiaries, %						
0-10	52 (19.8)	78 (29.8)	78 (29.8)	54 (20.6)	262 (100)	.01
11-20	96 (17.8)	186 (34.5)	144 (26.7)	113 (21.0)	539 (100)	
21-30	48 (17.1)	103 (36.8)	83 (29.6)	46 (16.4)	280 (100)	
>30	35 (26.7)	53 (40.5)	33 (25.2)	10 (7.6)	131 (100)	
Proportion of patients who are black						
Quartile 1, lowest proportion	52 (19.8)	78 (29.8)	78 (29.8)	54 (20.6)	262 (100)	<.001
Quartile 2	96 (17.8)	186 (34.5)	144 (26.7)	113 (21.0)	539 (100)	
Quartile 3	48 (17.1)	103 (36.8)	83 (29.6)	46 (16.4)	280 (100)	
Quartile 4, highest proportion	35 (26.7)	53 (40.5)	33 (25.2)	10 (7.6)	131 (100)	
Hospitals with 0 black patients	73 (22.0)	31 (9.3)	172 (51.8)	56 (16.9)	332 (100)	
Proportion of patients with low SES						
Quartile 1, lowest proportion	35 (13.4)	89 (34.1)	89 (34.1)	48 (18.4)	261 (100)	<.001
Quartile 2	39 (15.4)	92 (36.2)	70 (27.6)	53 (20.9)	254 (100)	
Quartile 3	44 (16.9)	126 (48.5)	34 (13.1)	56 (21.5)	260 (100)	
Quartile 4, highest proportion	76 (29.5)	94 (36.4)	65 (25.2)	23 (8.9)	258 (100)	
Hospitals with 0 patients with low SES	37 (20.7)	19 (10.6)	80 (44.7)	43 (24.0)	179 (100)	
Proportion of patients who are dually eligible for Medicare and Medicaid						
Quartile 1, lowest proportion	38 (13.7)	96 (34.7)	77 (27.8)	66 (23.8)	277 (100)	<.001
Quartile 2	46 (14.7)	95 (30.4)	105 (33.5)	67 (21.4)	313 (100)	
Quartile 3	53 (18.9)	101 (36.1)	70 (25.0)	56 (20.0)	280 (100)	
Quartile 4, highest proportion	93 (28.1)	127 (38.4)	77 (23.3)	34 (10.3)	331 (100)	
Hospitals with 0 dually eligible patients	1 (9.1)	1 (9.1)	9 (81.8)	0 (0.0)	11 (100)	
**Pneumonia**						
No. of hospitals	249	318	324	280	1171	NA
RSMR, median (IQR), %	10.0 (9.6-10.3)	9.8 (9.3-10.2)	13.5 (13.0-14.3)	13.5 (12.9-14.4)	NA	NA
RSP, median (IQR), $	12 763 (12 223-13 057)	15 815 (15 436-16 490)	12 653 (12 203-13 012)	15 781 (15 369-16 416)	NA	NA
Teaching status						
Teaching	30 (15.2)	106 (53.5)	16 (8.1)	46 (23.2)	198 (100)	<.001
Nonteaching	219 (22.5)	212 (21.8)	308 (31.7)	234 (24.0)	973 (100)	
Type of ownership						
Private for-profit	30 (15.5)	63 (32.5)	42 (21.6)	59 (30.4)	194 (100)	<.001
Private not-for-profit	170 (23.8)	226 (31.7)	174 (24.4)	144 (20.2)	714 (100)	
Public	49 (18.6)	29 (11.0)	108 (41.1)	77 (29.3)	263 (100)	
Safety-net status						
Safety-net hospital	69 (19.8)	49 (14.1)	143 (41.1)	87 (25.0)	348 (100)	<.001
Non–safety-net hospital	180 (21.9)	269 (32.7)	181 (22.0)	193 (23.5)	823 (100)	
Urban/rural status						
Urban	172 (20.2)	285 (33.5)	187 (22.0)	206 (24.2)	850 (100)	<.001
Rural	77 (24.0)	33 (10.3)	137 (42.7)	74 (23.1)	321 (100)	
No. of beds						
1-199	203 (24.5)	150 (18.1)	297 (35.9)	177 (21.4)	827 (100)	<.001
200-299	24 (15.8)	65 (42.8)	18 (11.8)	45 (29.6)	152 (100)	
300-399	9 (11.1)	36 (44.4)	3 (3.7)	33 (40.7)	81 (100)	
400-499	8 (17.8)	21 (46.7)	5 (11.1)	11 (24.4)	45 (100)	
≥500	5 (7.6)	46 (69.7)	1 (1.5)	14 (21.2)	66 (100)	
Proportion of patients who are Medicaid beneficiaries, %						
0-10	60 (20.6)	72 (24.7)	71 (24.4)	88 (30.2)	291 (100)	.18
11-20	114 (21.6)	146 (27.7)	150 (28.4)	118 (22.3)	528 (100)	
21-30	51 (20.5)	67 (26.9)	72 (28.9)	59 (23.7)	249 (100)	
>30	24 (23.3)	33 (32.0)	31 (30.1)	15 (14.6)	103 (100)	
Proportion of patients who are black						
Quartile 1, lowest proportion	40 (23.3)	46 (26.7)	54 (31.4)	32 (18.6)	172 (100)	<.001
Quartile 2	36 (18.8)	70 (36.5)	41 (21.4)	45 (23.4)	192 (100)	
Quartile 3	21 (10.5)	83 (41.5)	31 (15.5)	65 (32.5)	200 (100)	
Quartile 4, highest proportion	39 (19.3)	69 (34.2)	38 (18.8)	56 (27.7)	202 (100)	
Hospitals with 0 black patients	113 (27.9)	50 (12.3)	160 (39.5)	82 (20.2)	405 (100)	
Proportion of patients with low SES						
Quartile 1, lowest proportion	68 (27.8)	62 (25.3)	57 (23.3)	58 (23.7)	245 (100)	<.001
Quartile 2	48 (18.5)	74 (28.6)	72 (27.8)	65 (25.1)	259 (100)	
Quartile 3	46 (19.1)	86 (35.7)	41 (17.0)	68 (28.2)	241 (100)	
Quartile 4, highest proportion	50 (18.7)	71 (26.6)	91 (34.1)	55 (20.6)	267 (100)	
Hospitals with 0 patients with low SES	37 (23.3)	25 (15.7)	63 (39.6)	34 (21.4)	159 (100)	
Proportion of patients who are dually eligible for Medicare and Medicaid						
Quartile 1, lowest proportion	71 (26.7)	81 (30.5)	57 (21.4)	57 (21.4)	266 (100)	.04
Quartile 2	62 (22.4)	70 (25.3)	76 (27.4)	69 (24.9)	277 (100)	
Quartile 3	58 (19.8)	74 (25.3)	84 (28.7)	77 (26.3)	293 (100)	
Quartile 4, highest proportion	57 (17.6)	92 (28.4)	100 (30.9)	75 (23.1)	324 (100)	
Hospitals with 0 dually eligible patients	1 (9.1)	1 (9.1)	7 (63.6)	2 (18.2)	11 (100)	

Abbreviations: IQR, interquartile range; NA, not applicable; RSMRs, risk-standardized mortality rates; RSPs, risk-standardized payments; SES, socioeconomic status.

^a^ Values are inflation adjusted to 2013 US dollars. For acute myocardial infarction, 1319 of 1332 hospitals (99.0%) were matched to American Heart Association data. For heart failure, 1212 of 1228 hospitals (98.7%) were matched to American Heart Association data. For pneumonia, 1171 of 1199 hospitals (97.7%) were matched to American Heart Association data. Percentages may not sum to 100 because of rounding. Data are number (percentage) unless otherwise indicated.

## Discussion

In this national study of the value of AMI, HF, and PNA care based on the CMS publicly reported risk-standardized mortality and payment measures, we found a statistically significant but weak inverse correlation between RSMR and RSP for all 3 conditions, with a maximum shared variance of less than 5% that was consistent across hospital characteristics, suggesting substantial opportunities to improve the efficiency and value of care. In addition, we found that high-value care for AMI, HF, and PNA, defined by having both a low RSMR and low RSP, was achieved across diverse hospital types serving diverse patient populations, including those caring for a disproportionate share of patients with low SES.

A key finding of our study is the significant inverse but modest association between RSMR and RSP for AMI, HF, and PNA. The inverse correlation between RMSR and RSP for AMI is consistent with a prior study^22^ from Ontario, Canada, which found that hospitals with higher spending intensity had lower rates of 30-day mortality and suggested that there are instances where greater resource use may lead to improved quality and outcomes (eg, reperfusion therapy and provision of guideline-indicated pharmacotherapy for patients with AMI). Similarly, a recent examination of Medicare beneficiaries with AMI found that patients admitted to hospitals with 30-day payments 1 SD ($1750) above the mean compared with those 1 SD below the mean had an associated approximately 0.7% absolute lower mortality.^23^ Our finding of a similar pattern for HF and PNA suggests that there may likely be an association, albeit modest, between the intensity of care and clinical outcomes for these medical conditions. This finding aligns with prior data from California that found an inverse association between spending and mortality for medical conditions,^24^ as well as a prior Medicare analysis observing that hospitals with lower risk-adjusted costs had slightly worse performance on process-based quality indicators.^25^ Taken together, these results suggest that in some instances efforts to reduce use may come at the expense of worse clinical outcomes.

More broadly, the lack of a strong association between RSMRs and RSPs for AMI, HF, and PNA highlights the opportunity to improve efficiency and value in health care and the policy rationale for alternative payment models. Given that comparable RSMRs for AMI, HF, and PNA are achieved across a broad range of RSPs, hospitals with higher RSPs may have substantial opportunities for savings, while preserving or improving outcomes. Prior work has shown that in many instances discretionary decisions, such as the use of intensive care services,^26^ postacute care,^27,28^ and others, may increase expenditures without translating into improvements in clinical outcomes. The Bundled Payments for Care Improvement Advanced program is a voluntary 90-day program, which commenced October 1, 2018. and has established target prices for 32 clinical episodes, including AMI, HF, and PNA. Hospitals that meet requisite quality metrics and are able to deliver care under the target price will reap financial gains. Rigorous examination of the effect of alternative payment models on cost and quality, potential unintended consequences, and mixed-methods analyses of strategies and care pathways implemented at high-performing centers and associated with high-value care will represent foundational knowledge to help guide future efforts.

Another key finding from our study is that, regardless of size, setting, teaching status, and safety-net status, hospitals were able to achieve low RSMRs and low RSPs, suggesting that high-value care for AMI, HF, and PNA can be provided across a variety of hospital types. Approximately 1 in 4 hospitals with the highest proportions of patients who are Medicaid beneficiaries, black, low SES, or dually eligible for Medicare and Medicaid achieved a low RSMR and low RSR for AMI, HF, and PNA. In contrast to the concerns raised in the aftermath of the Hospital Readmissions Reduction Program,^29,30^ which financially penalizes hospitals for excess readmissions—in which safety-net hospitals have been disproportionately subject to penalties compared with non–safety-net hospitals—our findings suggest that policy initiatives that integrate both domains of spending and outcomes would not systematically disadvantage particular groups of hospitals. The real-world influence of policy initiatives will warrant additional investigation, as does our finding that hospitals with low RSMRs and low RSPs for AMI, HF, and PNA had markedly lower use of nonacute inpatient services. This strategy has been found to be beneficial among patients undergoing lower joint replacement and may represent an important element for success under the Bundled Payments for Care Improvement Advanced program and other alternative payment models for general medical conditions.^31^

Our findings have important implications for clinicians, payers, and policy makers. There is a pressing need to develop and refine empirical definitions of value wherein payments and outcomes are explicitly linked. An Institute of Medicine report entitled “Best Care at Lower Cost: The Path to Continuously Learning Health Care in America”^4^ emphasized the importance of transparency in outcomes and use as a predicate to value-driven health care. The 30-day RSMR and RSP measures may be able to have an important role toward this end. In fact, the condition-specific mortality measures are already included as part of the hospital Value-Based Purchasing Program,^32^ and the AMI, HF, and PNA payment measures are publicly reported but have recently been removed from the hospital Value-Based Purchasing Program.^33^ The successful transition from volume-based to value-based payment models necessitates empirical assessments of value, and our analysis represents an important step in that direction. Relatedly, policy initiatives aimed at facilitating the transition to value-based health care should integrate both clinical outcomes and spending. We found that hospitals in the low RSMR: high RSP group were more likely to be larger, teaching hospitals, private ownership, non–safety-net hospitals, and urban hospitals, while hospitals in the high RSMR: low RSP group were more likely to be smaller, nonteaching hospitals, public ownership, safety-net hospitals, and rural hospitals. As such, facilities with low RSPs but high RSMRs may be perversely rewarded by programs exclusively or asymmetrically rewarding use. In fact, a recent examination of the introduction of the Medicare Spending per Beneficiary measure into the hospital Value-Based Purchasing Program^34^ found that 17% of low-quality (defined by performance below the median) hospitals received payment bonuses by virtue of having low spending. Bundled payment programs and other policy initiatives that place downward pressure on payment and use must integrate careful evaluation of the influence on care quality and clinical outcomes. The Bundled Payment for Care Improvement Advanced program integrates benchmarks for quality that must be met for financial rewards to be realized.^35^ Additional work examining which hospital characteristics, strategies, and enabling structures may be associated with high-value care would inform performance improvement activities, as well as the design of other policy initiatives. Our cross-sectional analysis provides insights into the modest association between RSMRs and RSPs and highlights the complexity of the association. Specifically, these data would suggest that some hospitals have to increase spending, while others may be able to decrease spending to maintain or improve outcomes. Future studies that can identify specific strategies and interventions (during the index hospitalization, as well as postacute care) to improve the value and efficiency will be essential to fostering delivery and payment reform.

### Limitations

There are several limitations of our study. First, our study is limited to patients in the fee-for-service Medicare program and may not be generalized to the care for other patients. Regardless, Medicare is the largest payer and has historically been a principal driver of payment reform. Second, we examined the association between 30-day payment and outcome. While this period reflects what has been used for public reporting and RSMR and RSP measures are being incorporated into payment programs, the influence of certain interventions may not be realized within 30 days. Additional analyses looking at the association between spending and longer-term outcomes should be undertaken. Third, we examined hospital payments rather than costs. While other investigations have used charges or accounting costs,^25^ the RSPs (adjusted for differences in case mix) reflect resource use across clinicians and over an episode of care and were specifically developed to facilitate joint assessment of outcomes and use, as done in this analysis. Fourth, our approach relied on stratifying hospitals based on the median RSMR and RSP. While this has been used in prior analyses and in other policy programs by the CMS,^36,37^ adjudicating value solely based on the 50th percentile of performance may not provide the most meaningful designation of value. Additional empirical work to define value and inform policy initiatives seeking to reward high-value care is needed. Fifth, both the RMSR and the RSP rely on administrative claims data for risk adjustment. Although there may be instances where administrative data alone are incapable of fully capturing clinical case mix, this method has been validated in prior work,^38^ and these measures have been endorsed by the National Quality Forum. Sixth, all of the analyses that were conducted were static, looking at cross-sectional associations between payment and mortality. As additional years of data are accrued and hospitals participate in alternative payment models, such as the Bundled Payments for Care Improvement Advanced program, it will be important to test whether and how reductions in use and spending can be achieved without compromising quality of care and outcomes.

## Conclusions

Our national study of the value of AMI, HF, and PNA care based on the CMS publicly reported RSMR and RSP measures suggests substantial opportunities to improve the efficiency and value of care. In addition, the findings herein indicate that high-value care for these conditions may be provided across a variety of hospital types.

## Notes

Supplement. **eTable 1.** Acute Myocardial Infarction Patient Characteristics and Comorbidities **eTable 2.** Heart Failure Patient Characteristics and Comorbidities **eTable 3.** Pneumonia Patient Characteristics and Comorbidities **eTable 4.** Detailed Payment Information for Acute Myocardial Infarction Overall and Stratified by Median RMSR and RSP **eTable 5.** Detailed Payment Information for Congestive Heart Failure Overall and Stratified by Median RMSR and RSP **eTable 6.** Detailed Payment Information for Pneumonia Overall and Stratified by Median RMSR and RSP
