# Supplementary material for: Variation in and Hospital Characteristics Associated With the Value of Care for Medicare Beneficiaries With Acute Myocardial Infarction, Heart Failure, and Pneumonia
Source: JAMA Netw Open. 2018 Oct 19;1(6):e183519. doi: 10.1001/jamanetworkopen.2018.3519 (PMC6324438; doi:10.1001/jamanetworkopen.2018.3519)
Supplement: Supplement. — eTable 1. Acute Myocardial Infarction Patient Characteristics and Comorbidities eTable 2. Heart Failure Patient Characteristics and Comorbidities eTable 3. Pneumonia Patient Characteristics and Comorbidities eTable 4. Detailed Payment Information for Acute Myocardial Infarction Overall and Stratified by Median RMSR and RSP eTable 5. Detailed Payment Information for Congestive Heart Failure Overall and Stratified by Median RMSR and RSP eTable 6. Detailed Payment Information for Pneumonia Overall and Stratified by Median RMSR and RSP [file jamanetwopen-1-e183519-s001.pdf]

Supplementary Online Content

Desai NR, Ott LS, George EJ, et al. Variation in and hospital characteristics associated with the value of care for Medicare beneficiaries with acute myocardial infarction, heart failure, and pneumonia. *JAMA Netw Open*. 2018:1(6):e183519. doi:10.1001/jamanetworkopen.2018.3519

eTable 1. Acute Myocardial Infarction Patient Characteristics and Comorbidities

eTable 2. Heart Failure Patient Characteristics and Comorbidities

eTable 3. Pneumonia Patient Characteristics and Comorbidities

eTable 4. Detailed Payment Information for Acute Myocardial Infarction Overall and Stratified by Median RMSR and RSP

eTable 5. Detailed Payment Information for Congestive Heart Failure Overall and Stratified by Median RMSR and RSP

eTable 6. Detailed Payment Information for Pneumonia Overall and Stratified by Median RMSR and RSP

This supplementary material has been provided by the authors to give readers additional information about their work.

© 2018 Desai NR et al. *JAMA Network Open*.

eTable 1. Acute Myocardial Infarction Patient Characteristics and Comorbidities[1]

Variable	Frequency (%)	High RSMR: High RSP	High RSMR: Low RSP	Low RSMR: High RSP	Low RSMR: Low RSP
Total patients	460,374	109,278	69,586	188,081	93,429
Mean age minus 65 (standard deviation)	13.8 (8.3)	13.5 (8.3)	14.2 (8.5)	13.9 (8.3)	13.7 (8.3)
Male	51.5	52.0	50.0	51.8	51.4
History of Percutaneous Transluminal Coronary Angioplasty (PTCA) (ICD-9 codes V45.82, 00.66, 36.06, 36.07)	16.9	17.4	15.4	17.6	16.1
History of Coronary Artery Bypass Graft (CABG) surgery (ICD-9 codes V45.81, 36.10-36.16)	11.8	12.1	11.5	12.0	11.4
Congestive heart failure (CC 80)	29.5	29.0	30.6	29.7	29.0
Acute myocardial infarction (CC 81)	13.0	12.3	13.1	13.0	13.7
Other acute/subacute forms of ischemic heart disease (CC 82)	13.0	12.6	12.6	13.5	12.9
Anterior myocardial infarction (ICD-9 codes 410.00-410.12)	8.2	8.5	7.5	8.3	8.1
Other location of myocardial infarction (ICD-9 codes 410.20-410.62)	12.4	13.1	11.4	12.4	12.3
Coronary atherosclerosis or angina (CC 83-84)	85.0	86.2	81.2	86.2	84.3
Cardio-respiratory failure or shock (CC 79)	10.3	10.3	10.4	10.4	10.0
Valvular or rheumatic heart disease (CC 86)	31.4	30.7	29.5	32.5	31.4
Hypertension (CC 89, 91)	89.0	88.9	89.2	89.2	88.9
Stroke (CC 95-96)	7.2	7.3	7.3	7.3	6.9
Cerebrovascular disease (CC 97-	20.6	20.9	20.1	20.9	19.9

[1] Total patients included in both the mortality and payment measure cohort.

ICD-9 codes are the International Classification of Diseases, Ninth Revision, Clinical Modification (ICD-9-CM), based on the World Health Organization's Ninth Revision, International Classification of Diseases (ICD-9). CC codes are CMS Condition Categories. These are groupings of ICD-9-CM diagnosis codes in clinically relevant categories, from the Hierarchical Condition Category (HSS) system [1,2]. The risk variables are created using the grouping but not the hierarchical logic of the system.

[1]. Pope G, Ellis R, Ash A, et al. Diagnostic Cost Group Hierarchical Condition Category Models for Medicare Risk Adjustment. Final Report to the Health Care Financing Administration under Contract Number 500-95-048. 2000. Available at http://www.cms.hhs.gov/Reports/downloads/pope_2000_2.pdf. Accessed July 19 2018.

[2]. Pope G, Kautter J, Ingber M, et al. Evaluation of the CMS-HCC Risk Adjustment Model: Final Report. 2011. Available at https://www.cms.gov/Medicare/Health-Plans/MedicareAdvtgSpecRateStats/downloads/evaluation_risk_adj_model_2011.pdf. Accessed July 19, 2018.

© 2018 Desai NR et al. *JAMA Network Open*.

Variable	Frequency (%)	High RSMR: High RSP	High RSMR: Low RSP	Low RSMR: High RSP	Low RSMR: Low RSP
99, 103)					
Renal failure (CC 131)	26.1	25.7	26.7	26.4	25.7
Chronic obstructive pulmonary disease (COPD) (CC 108)	30.3	31.2	31.0	29.6	30.1
Pneumonia (CC 111-113)	22.7	22.7	23.5	22.9	21.8
Diabetes mellitus (DM) or DM complications except proliferative retinopathy (CC 15-20, 120)	46.7	47.0	46.6	46.7	46.2
Protein-calorie malnutrition (CC 21)	6.3	5.9	6.0	6.6	6.5
Dementia or other specified brain disorders (CC 49-50)	19.8	19.5	21.4	19.8	19.0
Hemiplegia, paraplegia, paralysis, functional disability (CC 67-69, 100-102, 177-178)	6.3	6.4	6.5	6.4	6.1
Vascular disease and complications (CC 104-105)	27.2	27.3	25.6	28.3	26.1
Metastatic cancer, acute leukemia and other severe cancers (CC 7-8)	3.8	3.7	3.6	3.9	3.7
Trauma in last year (CC 154-156, 158-162)	31.4	31.1	31.8	31.8	30.6
Major psychiatric disorders (CC 54-56)	7.9	7.7	8.2	8.1	7.6
Chronic liver disease (CC 25-27)	1.5	1.5	1.3	1.5	1.4

© 2018 Desai NR et al. *JAMA Network Open*.

eTable 2. Heart Failure Patient Characteristics and Comorbidities[2]

Variable	Frequency (%)	High RSMR: High RSP	High RSMR: Low RSP	Low RSMR: High RSP	Low RSMR: Low RSP
Total patients	883,970	236,426	145,198	371,571	130,775
Mean age minus 65 (standard deviation)	16.1 (8.3)	16.1 (8.2)	16.1 (8.3)	16.2 (8.3)	15.9 (8.3)
Male	44.5	45.3	43.3	44.9	43.1
History of Percutaneous Transluminal Coronary Angioplasty (PTCA) (ICD-9 codes V45.82, 00.66, 36.06, 36.07)	13.5	14.3	10.9	14.6	11.8
History of Coronary Artery Bypass Graft (CABG) surgery (ICD-9 codes V45.81, 36.10-36.16)	18.9	19.8	16.8	19.8	17.2
Congestive heart failure (CC 80)	73.6	72.8	72.2	74.6	73.6
Acute myocardial infarction (CC 81)	9.5	9.3	8.8	10.0	9.1
Other acute/subacute forms of ischemic heart disease (CC 82)	12.1	11.6	10.6	13.2	11.4
Coronary atherosclerosis or angina (CC 83-84)	72.6	72.8	68.0	75.1	69.9
Cardio-respiratory failure or shock (CC 79)	26.5	26.7	25.0	27.4	25.3
Valvular or rheumatic heart disease (CC 86)	53.2	53.6	47.7	56.3	49.9
Hypertension (CC 89, 91)	93.5	93.4	92.9	93.8	93.7
Stroke (CC 95-96)	9.3	9.0	8.3	10.0	8.8
Renal failure (CC 131)	49.3	48.5	46.5	51.1	48.9
Chronic obstructive pulmonary disease (COPD) (CC 108)	48.3	48.3	48.2	48.0	49.1
Pneumonia (CC 111-113)	45.0	44.4	43.2	46.1	44.5
Diabetes mellitus (DM) or DM complications except proliferative	53.8	52.8	52.3	54.9	53.8

[2] Total patients included in both the mortality and payment measure cohort.
ICD-9 codes are the International Classification of Diseases, Ninth Revision, Clinical Modification (ICD-9-CM), based on the World Health Organization's Ninth Revision, International Classification of Diseases (ICD-9). CC codes are CMS Condition Categories. These are groupings of ICD-9-CM diagnosis codes in clinically relevant categories, from the Hierarchical Condition Category (HSS) system [1,2]. The risk variables are created using the grouping but not the hierarchical logic of the system.
[1]. Pope G, Ellis R, Ash A, et al. Diagnostic Cost Group Hierarchical Condition Category Models for Medicare Risk Adjustment. Final Report to the Health Care Financing Administration under Contract Number 500-95-048. 2000. Available at http://www.cms.hhs.gov/Reports/downloads/pope_2000_2.pdf. Accessed July 19 2018.
[2]. Pope G, Kautter J, Ingber M, et al. Evaluation of the CMS-HCC Risk Adjustment Model: Final Report. 2011. Available at https://www.cms.gov/Medicare/Health-Plans/MedicareAdvtgSpecRateStats/downloads/evaluation_risk_adj_model_2011.pdf. Accessed July 19, 2018.

© 2018 Desai NR et al. *JAMA Network Open*.

Variable	Frequency (%)	High RSMR: High RSP	High RSMR: Low RSP	Low RSMR: High RSP	Low RSMR: Low RSP
retinopathy (CC 15-20, 120)					
Protein-calorie malnutrition (CC 21)	10.1	9.1	8.7	11.3	10.2
Dementia or other specified brain disorders (CC 49-50)	24.9	24.0	24.1	25.7	24.8
Hemiplegia, paraplegia, paralysis, functional disability (CC 67-69, 100-102, 177-178)	8.5	8.2	7.9	9.0	8.4
Vascular disease and complications (CC 104-105)	38.4	37.4	33.4	41.7	36.0
Metastatic cancer, acute leukemia and other severe cancers (CC 7-8)	4.4	4.3	4.0	4.7	4.1
Trauma in last year (CC 154-156, 158-162)	40.4	40.5	39.3	41.0	39.5
Major psychiatric disorders (CC 54-56)	10.7	10.5	10.1	11.2	10.3
Chronic liver disease (CC 25-27)	3.1	2.8	2.6	3.5	2.9

© 2018 Desai NR et al. *JAMA Network Open*.

eTable 3. Pneumonia Patient Characteristics and Comorbidities[3]

Variable	Frequency (%)	High RSMR: High RSP	High RSMR: Low RSP	Low RSMR: High RSP	Low RSMR: Low RSP
Total patients	895,016	238,725	168,633	304,874	182,784
Mean age minus 65 (standard deviation)	15.3 (8.4)	15.2 (8.3)	15.3 (8.4)	15.5 (8.4)	15.3 (8.4)
Male	44.6	44.5	44.9	44.5	44.6
History of Percutaneous Transluminal Coronary Angioplasty (PTCA) (ICD-9 codes V45.82, 00.66, 36.06, 36.07)	7.6	7.8	6.7	8.0	7.3
History of Coronary Artery Bypass Graft (CABG) surgery (ICD-9 codes V45.81, 36.10-36.16)	9.3	9.5	8.6	9.7	9.1
Congestive heart failure (CC 80)	38.3	38.2	37.3	39.1	38.0
Acute myocardial infarction (CC 81)	3.9	3.9	3.5	4.1	3.7
Other acute/subacute forms of ischemic heart disease (CC 82)	5.8	5.8	5.0	6.4	5.6
Coronary atherosclerosis or angina (CC 83-84)	49.5	49.7	46.9	51.3	48.5
Cardio-respiratory failure or shock (CC 79)	21.8	22.3	19.7	23.0	20.9
Hypertension (CC 89, 91)	87.3	87.5	86.3	87.9	87.0
Stroke (CC 95-96)	8.9	9.2	8.2	9.3	8.4
Cerebrovascular disease (CC 97-99, 103)	21.7	22.1	20.2	22.7	20.9
Renal failure (CC 131)	29.8	29.8	27.5	31.3	29.4
Chronic obstructive pulmonary disease (COPD) (CC 108)	54.3	55.1	54.7	53.3	54.4
Pneumonia (CC 111-113)	41.1	41.4	40.5	41.5	40.3
Protein-calorie malnutrition (CC 21)	12.8	12.5	10.6	14.1	13.0

[3] Total patients included in both the mortality and payment measure cohort.
ICD-9 codes are the International Classification of Diseases, Ninth Revision, Clinical Modification (ICD-9-CM), based on the World Health Organization's Ninth Revision, International Classification of Diseases (ICD-9). CC codes are CMS Condition Categories. These are groupings of ICD-9-CM diagnosis codes in clinically relevant categories, from the Hierarchical Condition Category (HSS) system [1,2]. The risk variables are created using the grouping but not the hierarchical logic of the system.

[1]. Pope G, Ellis R, Ash A, et al. Diagnostic Cost Group Hierarchical Condition Category Models for Medicare Risk Adjustment. Final Report to the Health Care Financing Administration under Contract Number 500-95-048. 2000. Available at http://www.cms.hhs.gov/Reports/downloads/pope_2000_2.pdf. Accessed July 19 2018.
[2]. Pope G, Kautter J, Ingber M, et al. Evaluation of the CMS-HCC Risk Adjustment Model: Final Report. 2011. Available at https://www.cms.gov/Medicare/Health-Plans/MedicareAdvtgSpecRateStats/downloads/evaluation_risk_adj_model_2011.pdf. Accessed July 19, 2018.

© 2018 Desai NR et al. *JAMA Network Open*.

Variable	Frequency (%)	High RSMR: High RSP	High RSMR: Low RSP	Low RSMR: High RSP	Low RSMR: Low RSP
Dementia or other specified brain disorders (CC 49-50)	30.7	30.8	30.3	31.2	29.9
Hemiplegia, paraplegia, paralysis, functional disability (CC 67-69, 100-102, 177-178)	8.7	8.8	8.3	9.1	8.4
Vascular disease and complications (CC 104-105)	31.4	31.1	28.0	34.1	30.3
Metastatic cancer, acute leukemia, and other severe cancers (CC 7-8)	9.2	9.1	8.1	10.1	8.9
Trauma in last year (CC 154-156, 158-162)	40.7	40.8	39.8	41.5	40.2
Major psychiatric disorders (CC 54-56)	13.8	14.0	13.3	14.4	13.1
Chronic liver disease (CC 25-27)	2.1	2.0	1.8	2.4	2.0
Severe hematological disorders (CC 44)	2.7	2.6	2.2	3.1	2.5
Parkinson's or Huntington's diseases (CC 73)	4.0	4.1	3.9	4.1	3.8
Fibrosis of lung or other chronic lung disorders (CC 109)	14.6	14.5	13.1	15.7	14.4
Iron deficiency or other unspecified anemias and blood disease (CC 47)	58.0	57.9	55.3	60.2	57.2
Depression (CC 58)	24.6	24.2	24.5	24.6	25.3
Seizure disorders and convulsions (CC 74)	5.7	5.9	5.3	6.0	5.4
Asthma (CC 110)	11.8	11.3	11.0	12.6	11.8
Vertebral fractures (CC 157)	5.1	5.1	4.9	5.3	5.1

© 2018 Desai NR et al. *JAMA Network Open*.

eTable 4. Detailed Payment Information for Acute Myocardial Infarction Overall and Stratified by Median RMSR and RSP

Care Setting/Service	Overall	High RSMR: High RSP	High RSMR: Low RSP	Low RSMR: High RSP	Low RSMR: Low RSP
**Index Hospitalization**					
# of patients	444,105	106,528	63,141	184,856	89,580
Median (IQR), $	12448 (10025 - 18700)	12677 (10582 - 19587)	11837 (7936 - 14149)	12638 (10546 - 19405)	12058 (8442 - 15246)
**Post-Acute Care (total)**					
# of patients	387,775	91,384	53,469	164,042	78,880
Median (IQR), $	1948 (364 - 9202)	2208 (377 - 9983)	1656 (324 - 8170)	2113 (396 - 9707)	1515 (320 - 7799)
**Readmission (Facility and Physician)**					
# of patients	153,978	38,645	20,134	66,378	28,821
Median (IQR), $	2060 (634 - 5871)	2052 (649 - 5874)	1933 (579 - 5871)	2115 (672 - 5861)	2022 (587 - 5861)
**Skilled Nursing Facilities**					
# of patients	90,874	22,150	11,825	39,945	16,954
Median (IQR), $	7895 (3624 - 11438)	7911 (3715 - 11421)	7505 (3261 - 11134)	8073 (3720 - 11623)	7706 (3555 - 11151)
**Hospice**					
# of patients	15,806	4,100	2,579	6,145	2,982
Median (IQR), $	2440 (1197 - 3910)	2470 (1214 - 4001)	2321 (1077 - 3844)	2406 (1197 - 3899)	2462 (1137 - 3889)
**Home Health Agency**					
# of patients	87,735	20,568	10,895	39,415	16,857
Median (IQR)	1109 (490 - 1891)	1092 (479 - 1890)	1090 (486 - 1864)	1123 (484 - 1903)	1107 (516 - 1877)
**Non-Acute Inpatient Settings**					
# of patients	18,548	5,340	1,984	8,590	2,634
Median (IQR), $	15137 (11043 - 20066)	15504 (11298 - 20082)	14722 (9897 - 19440)	15301 (11257 - 20185)	14569 (10739 - 19548)
**Observation Stay**					
# of patients	17,250	4,242	2,433	7,093	3,482
Median (IQR), $	1249 (181 - 1946)	1222 (181 - 1959)	1230 (181 - 1944)	1258 (199 - 1936)	1271 (181 - 1946)
**Emergency Department**					

© 2018 Desai NR et al. *JAMA Network Open*.

# of patients	89,377	21,538	12,474	37,441	17,924
$ per patient	236 (177 - 591)	231 (178 - 573)	246 (177 - 629)	231 (177 - 565)	243 (177 - 637)
**Outpatient Physician Visits**					
# of patients	259,486	60,681	36,076	109,862	52,867
Median (IQR), $	182 (105 - 313)	182 (105 - 314)	176 (105 - 297)	195 (105 - 323)	176 (105 - 295)
**Other Outpatient Settings**					
# of patients	241,824	55,303	33,565	102,119	50,837
Median (IQR), $	100 (33 - 337)	97 (31 - 344)	97 (32 - 330)	101 (34 - 337)	102 (35 - 334)
**Miscellaneous**					
# of patients	160,721	38,307	21,523	69,301	31,590
Median (IQR), $	300 (147 - 577)	300 (174 - 603)	300 (141 - 544)	300 (150 - 576)	300 (135 - 560)

© 2018 Desai NR et al. *JAMA Network Open*.

eTable 5. Detailed Payment Information for Congestive Heart Failure Overall and Stratified by Median RMSR and RSP

Care Setting/Service	Overall	High RSMR: High RSP	High RSMR: Low RSP	Low RSMR: High RSP	Low RSMR: Low RSP
**Index Hospitalization**					
# of patients	871,246	233,376	141,722	368,782	127,366
Median (IQR), $	7309 (6246 - 9776)	7432 (6283 - 9812)	6733 (6002 - 9299)	7770 (6414 - 10163)	6802 (6057 - 9363)
**Post-Acute Care (total)**					
# of patients	814,278	217,465	130,760	346,597	119,456
Median (IQR), $	2829 (644 - 10566)	3084 (698 - 11126)	2458 (542 - 9231)	3007 (709 - 11198)	2368 (527 - 9099)
**Readmission (Facility and Physician)**					
# of patients	331,486	89,072	48,437	147,785	46,192
Median (IQR), $	2322 (696 - 6060)	2260 (694 - 6126)	1942 (546 - 5949)	2539 (810 - 6203)	2026 (584 - 5904)
**Skilled Nursing Facilities**					
# of patients	248,240	68,852	39,259	106,369	33,760
Median (IQR), $	8395 (3617 - 11884)	8627 (3840 - 12067)	7947 (3335 - 11390)	8558 (3735 - 12093)	7895 (3178 - 11401)
**Hospice**					
# of patients	55,091	16,652	9,906	20,952	7,581
Median (IQR), $	2393 (1105 - 3770)	2406 (1172 - 3825)	2244 (1047 - 3694)	2393 (1203 - 3825)	2502 (1197 - 3756)
**Home Health Agency**					
# of patients	235,272	61,405	33,418	107,836	32,613
Median (IQR)	1052 (447 - 1820)	1073 (457 - 1854)	1088 (489 - 1852)	1028 (413 - 1794)	1054 (466 - 1795)
**Non-Acute Inpatient Settings**					
# of patients	28,426	8,885	2,370	14,908	2,263
Median (IQR), $	16438 (10858 - 22177)	16685 (11569 - 21766)	15074 (8779 - 20778)	16820 (11121 - 22986)	14807 (8608 - 20585)
**Observation Stay**					
# of patients	26,256	7,006	4,635	10,516	4,099
Median (IQR), $	1247 (181 - 1865)	1240 (181 - 1884)	1292 (203 - 1869)	1222 (181 - 1850)	1281 (202 - 1891)
**Emergency Department**					

© 2018 Desai NR et al. *JAMA Network Open*.

# of patients	189,532	50,983	30,628	79,883	28,038
$ per patient	229 (177 - 569)	235 (177 - 594)	267 (176 - 668)	226 (177 - 486)	239 (175 - 625)
**Outpatient Physician Visits**					
# of patients	515,186	138,895	81,896	220,023	74,372
Median (IQR), $	198 (105 - 333)	200 (105 - 334)	177 (105 - 309)	209 (105 - 350)	179 (105 - 314)
**Other Outpatient Settings**					
# of patients	545,940	145,465	89,236	228,946	82,293
Median (IQR), $	97 (33 - 322)	96 (32 - 318)	92 (32 - 292)	100 (33 - 347)	93 (33 - 303)
**Miscellaneous**					
# of patients	453,159	118,663	70,318	199,508	64,670
Median (IQR), $	296 (165 - 502)	296 (168 - 502)	281 (174 - 496)	296 (166 - 521)	281 (147 - 496)

© 2018 Desai NR et al. *JAMA Network Open*.

eTable 6. Detailed Payment Information for Pneumonia Overall and Stratified by Median RMSR and RSP

Care Setting/Service	Overall	High RSMR: High RSP	High RSMR: Low RSP	Low RSMR: High RSP	Low RSMR: Low RSP
**Index Hospitalization**					
# of patients	887,983	236,948	166,783	303,281	180,971
Median (IQR), $	6678 (5971 - 9334)	6892 (6029 - 9507)	6315 (5563 - 8851)	7121 (6124 - 9682)	6440 (5881 - 9034)
**Post-Acute Care (total)**					
# of patients	814,584	215,990	150,197	281,599	166,798
Median (IQR), $	2651 (479 - 10602)	3059 (533 - 11502)	2126 (386 - 9055)	3018 (578 - 11529)	2168 (399 - 8979)
**Readmission (Facility and Physician)**					
# of patients	285,717	79,934	48,260	103,545	53,978
Median (IQR), $	2064 (610 - 6194)	1994 (598 - 6133)	1779 (485 - 6165)	2306 (740 - 6307)	1886 (544 - 6052)
**Skilled Nursing Facilities**					
# of patients	284,453	79,431	50,389	101,249	53,384
Median (IQR), $	8269 (3334 - 11912)	8587 (3618 - 12209)	7616 (2890 - 11295)	8604 (3597 - 12242)	7768 (2972 - 11430)
**Hospice**					
# of patients	44,605	13,325	8,573	14,575	8,132
Median (IQR), $	2148 (970 - 3590)	2139 (980 - 3609)	2041 (915 - 3399)	2189 (1053 - 3657)	2199 (1047 - 3590)
**Home Health Agency**					
# of patients	190,735	49,517	30,587	73,251	37,380
Median (IQR)	1111 (449 - 1939)	1087 (441 - 1920)	1109 (473 - 1926)	1099 (437 - 1932)	1162 (478 - 1984)
**Non-Acute Inpatient Settings**					
# of patients	31,300	10,830	2,779	14,254	3,437
Median (IQR), $	17355 (11228 - 24758)	17710 (11878 - 24784)	14737 (8248 - 21994)	18163 (11934 - 26116)	15056 (8202 - 21496)
**Observation Stay**					
# of patients	20,095	5,135	4,062	6,722	4,176
Median (IQR), $	1352 (217 - 2033)	1323 (213 - 2024)	1374 (247 - 2051)	1339 (214 - 1994)	1387 (247 - 2078)
**Emergency Department**					
# of patients	163,817	44,042	30,268	56,400	33,107

© 2018 Desai NR et al. *JAMA Network Open*.

$ per patient	240 (176 - 598)	238 (177 - 597)	295 (176 - 695)	229 (177 - 531)	250 (175 - 623)
**Outpatient Physician Visits**					
# of patients	472,907	124,379	86,443	163,881	98,204
Median (IQR), $	173 (105 - 303)	173 (105 - 306)	152 (104 - 277)	176 (105 - 318)	163 (105 - 290)
**Other Outpatient Settings**					
# of patients	488,785	126,814	91,622	167,216	103,133
Median (IQR), $	84 (27 - 300)	81 (27 - 307)	77 (27 - 269)	88 (28 - 322)	85 (28 - 292)
**Miscellaneous**					
# of patients	457,734	121,504	81,487	163,767	90,976
Median (IQR), $	293 (153 - 489)	296 (166 - 496)	266 (140 - 489)	296 (174 - 496)	271 (133 - 484)

© 2018 Desai NR et al. *JAMA Network Open*.
